# *Entamoeba histolytica*: Diagnostic Microscopy in Clinical Amoebiasis

**DOI:** 10.4269/ajtmh.26-0105

**Published:** 2026-03-17

**Authors:** Devyani Sharma, Divya Rattan, Rakesh Sehgal

**Affiliations:** ^1^ Department of Parasitology, PGIMER, Chandigarh, India;; ^2^ Aarupadai Veedu Medical College and Hospital, Vinayaka Missions Research Foundation, Puducherry, India

## INTRODUCTION

**Figure 1.**
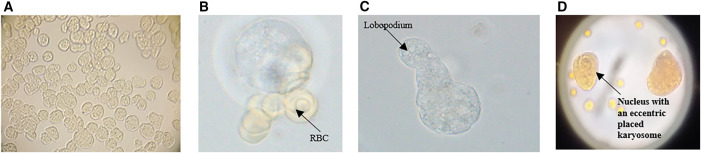
*Entamoeba histolytica* trophozoites from an axenic culture (**A–D**; 40× magnification). (**A**) trophozoites; (**B**) trophozoite exhibiting erythrophagocytosis; (**C**) single trophozoite exhibiting amoeboid movement and lobopodium; (**D**) iodine stain. The scale bar for all the images is 5 *µ*m.

**Figure 2.**
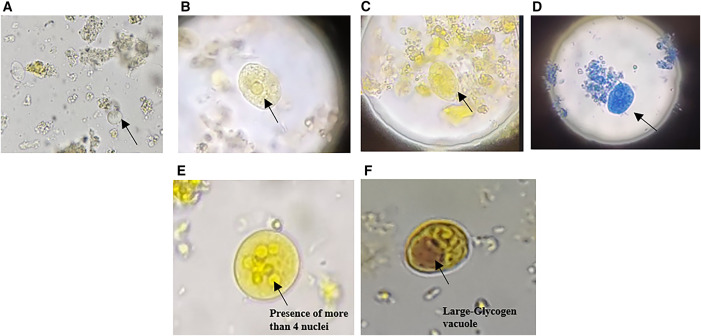
Patient stool sample with trophozoites of *Entamoeba* species (species-level identification is not possible using microscopy alone; 40× magnification). (**A**) Wet mount, (**B** and **C**) iodine mount, (**D**) methylene blue stain, (**E**) *Entamoeba coli*, (**F**) *Iodamoeba butschlii*. The scale bar for all the images is 10 *µ*m.

**Figure 3.**
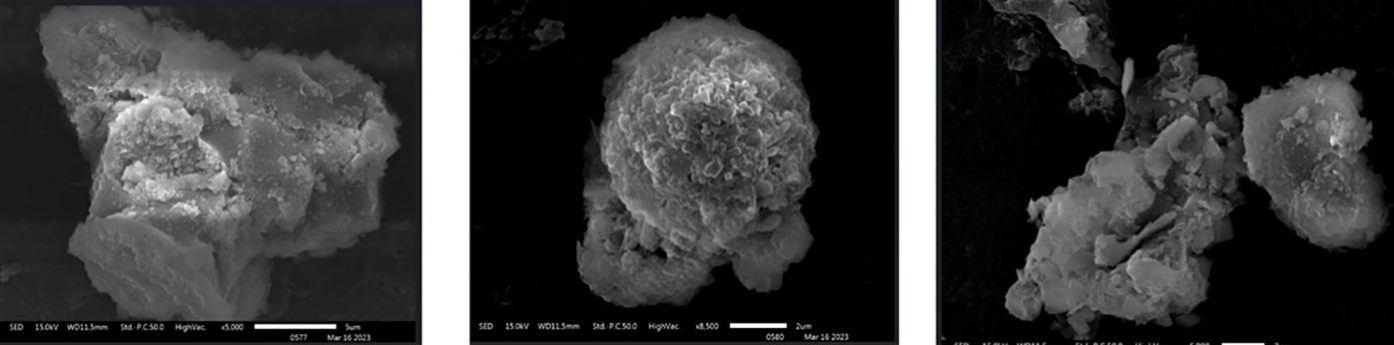
Scanning electron micrographs showing (**A** and **B**) *Entamoeba histolytica* trophozoite (axenic culture) with visible lobopodium. (**C**) *Entamoeba* species trophozoite (stool sample).

*Entamoeba histolytica* (*E. histolytica*) remains a major cause of invasive intestinal disease in tropical regions. Although the *Entamoeba* genus comprises numerous commensal species, recent studies have suggested that *Entamoeba dispar* (*E. dispar*) and *Entamoeba moshkovskii* (*E. moshkovskii*) may also induce gastrointestinal symptoms.^1^ Here, the microscopic visualization of trophozoites from standard axenic culture HM1:IMSS (Human isolate, Instituto Mexicano del Seguro Social) and a patient sample are presented using different stains and scanning electron microscopy (SEM) (Figures 1–3).^2^^,^^3^ A male patient presented to the out patient department with a 14-day history of abdominal pain and loose stools. A same-day stool examination revealed trophozoites of an *E. histolytica/E. dispar/E. moshkovskii* complex, but no other intestinal parasites were detected (Figure 2). The patient was started on anti-amoebic therapy. Erythrophagocytosis was observed in cultured trophozoites, a feature strongly associated with invasive *E. histolytica*. Scanning electron microscope images revealed prominent lobopodial extensions (Figure 3). Polymerase chain reaction testing confirmed infection with *E. histolytica*.^4^

In the authors’ experience, iodine mount and methylene blue stain are better suited for identifying trophozoites, despite reduced motility compared with wet mount (Figure 2). *Entamoeba coli* and *Iodamoeba butschlii*, which are often mistaken for *E. histolytica* on routine microscopy, are shown in Figure 2. Although SEM is not used in routine clinical diagnosis, it is presented here for morphological illustration. Although microscopy alone cannot differentiate among the three species, it remains essential for rapid detection in endemic regions. Overreliance on advanced diagnostic erodes these critical microscopy skills, risking patient care and public health.^5^
